# A 7-Year Report of Spectrum of Inborn Errors of Metabolism on Full-Term and Premature Infants in a Chinese Neonatal Intensive Care Unit

**DOI:** 10.3389/fgene.2019.01302

**Published:** 2020-01-10

**Authors:** Wanqiao Zhang, Yao Yang, Wei Peng, Juan Chang, Yabo Mei, Lei Yan, Yuhan Chen, Xiujuan Wei, Yabin Liu, Yan Wang, Zhichun Feng

**Affiliations:** ^1^ BaYi Children’s Hospital, Seventh Medical Center of PLA General Hospital, Beijing, China; ^2^ National Engineering Laboratory for Birth Defects Prevention and Control of Key Technology, Beijing, China; ^3^ Beijing Key Laboratory of Pediatric Organ Failure, Beijing, China

**Keywords:** newborn screening, neonatal intensive care unit, inborn errors of metabolism, incidence of inborn errors of metabolism, spectrum of genes and mutations

## Abstract

Inborn errors of metabolism (IEMs) have great repercussions in neonatal intensive care units (NICUs). However, the integrative analysis of the incidence for full-term and premature neonates of IEMs in NICUs have not been reported. In this study, we aimed to estimate the incidence of IEMs in the NICU population so as to better evaluate the impact of IEMs on Chinese NICUs. A total of 42,257 newborns (proportion of premature as 36.7%) enrolled to the largest Chinese NICU center for a sequential 7 years screen, and 66 were diagnosed with IEMs. The prevalence of IEMs in total, full-term, and premature infants was 1:640, 1:446, and 1:2,584, respectively. In spectrum of our NICU, diseases that cause endogenous intoxication like methylmalonic acidemia accounted for 93.9% (62/66), and this ratio was higher in full-term infants with 98.3% (59/60), while the most prevalent disease in premature newborn was hyperphenylalaninemia (50%, 3/6), respectively. The genetic analysis of 49 cases revealed 62 potentially pathogenic mutations in 10 well-documented pathogenic genes of IEMs, among which 21 were novel. In conclusion, differences in incidence and spectrum of full-term and premature births we obtained in NICU will provide diagnostic guidelines and therapeutic clues of neonatal IEMs for pediatricians.

## Introduction

Inborn errors of metabolism (IEM) are a phenotypically and genetically heterogeneous group of disorders caused by a defective enzyme, cofactor, or transporter in a metabolic pathway, leading to metabolic malfunctioning and/or the accumulation of toxic intermediate/terminal metabolites. This group of disorders involve in metabolism of various substances, such as amino acid, organic acidemias, fatty acid, and so on. As new concepts and techniques become available for identifying biochemical phenotypes, the number of such disorders has been constantly increasing up to more than 1,000 to date (Mak et al., 2013). Though individually rare, the cumulative incidence is about 1:800 in live births (Pampols, 2010). IEMs can present at any age from newborns to the elders. The clinical spectrum of IEM is diversified, either multi-systemic or with single-organ involvement, which can lead to death or severe disabilities if in time intervention were not introduced.

Newborn screening (NBS) for IEMs can prevent death and disability through early diagnosis and pre symptomatic treatment, recognizing as a huge success in field of public health of the last 50 years (Wilcken and Wiley, 2015). With the development of multiple testing and applications of molecular testing, especially the tandem mass spectrometry (MS/MS) technology, NBS has been rapidly improved and widely spread worldwide (Ombrone et al., 2016). In China mainland, MS/MS screening for IEMs using dry blood spot (DBS) launched in 2004 (Han et al., 2008). At present, the strategy has been administrated unbiasedly for normal newborns in some developed regions, whereas subjected exclusively for infants with high-risk inclination known as selective screening in other areas. Conclusions drawn from different studies in this field are controversial due to different selection criteria and target groups. The incidence of IEMs on normal newborns in different cities and regions of China was from 1/1,683 to 1/8,304 (Guo et al., 2018). While in some studies on selective screening of high-risk children with IEMs, the incidence was as high as 1:16 (on 4,981 cases) (Han et al., 2008), 1:51 (on 6,210 cases) (Sun et al., 2011), or 1:53 (on 16,075 cases) (Jiang et al., 2015).

As about 25% of IEMs can have manifestations in the neonatal period, newborns requiring admission to the neonatal intensive care unit (NICU) due to severe symptoms or low survival ability should actively consider the effects of IEMs by general practitioners (Jouvet et al., 2007; Couce et al., 2011; El-Hattab, 2015). Despite few studies from 31 cases in Spain (Couce et al., 2011) and 724 cases in China (Tu et al., 2012) reporting the distribution of IEMs in the corresponding NICU, the evaluation of impacts for IEM in NICU are still largely unveiled. In this study, we aim to estimate the incidence and characteristics of IEMs in full-term and premature neonates in a NICU in mainland China based on the screening results of 42,257 newborns in 7 years. We discovered that the total incidence of IEMs in a Chinese NICU was 1:640; while that in full-term neonates was 1:446 and that in premature ones was 1:2,584. Among the nine kinds of IEMs diagnosed in our study, the most common disease in full-term or all NICU neonates was methylmalonic acidemia (MMA). We found 62 potentially pathogenic mutations (including 21 novel ones) in 10 well-documented pathogenic genes of diagnostic cases of IEMs, and the most prevalent defective gene was *MMUT*. Our results enriched the understanding of the characteristics and genetics of neonatal IEMs and provided related diagnostic and therapeutic clues for neonatal pediatricians.

## Materials and Methods

### Study Participants

All the newborn patients admitted to the NICU of the affiliated BaYi Children’s Hospital of Seventh Medical Center of PLA General Hospital between January 1, 2010, and December 31, 2016 were enrolled to conduct biochemical analysis using MS/MS and gas chromatography–mass spectrometry (GC-MS) to screen IEMs. The hospital is a medical center for neonates in North China, attending seriously ill newborns (aged between several hours and 30 days) with low survival ability. A total of 42,257 newborns admitted to the NICU, including 26,750 full-term infants (GA ≥ 37 weeks) and 15,507 premature infants (GA <37 weeks), were enrolled for the study (**Supplementary Table 1**). Informed consent was obtained from the patient’s guardians for experimentation. All experiments were conducted in adherence to standard biosecurity and institutional safety procedures. All procedures followed were in accordance with the ethical standards of the responsible committee on human experimentation (institutional and national) and with the Helsinki Declaration of 1975, as revised in 2000.

### Tandem Mass Spectrometry Analysis

Dried blood filter papers were collected from neonates fed more than 48 h, and transferred to metabolic screening laboratory in BaYi Children’s Hospital on the same day. Blood amino acids and acylcarnitines were analyzed with MS/MS using in butyl-derivatized specimens (Shigematsu et al., 2002). An API 3200 (Applied Biosystems, Foster City, CA, USA) was used for MS/MS.

### Gas Chromatography–Mass Spectrometry Analysis

Urine samples of the infants with primary positive result in MS/MS analysis were taken to test the metabolic profiles using GC-MS. Urine metabolome analysis with urease-pretreatment was conducted as reported previously (Kuhara, 2002). A “TRACE GC ULTRA-ISQ” instrument (Thermo Fisher Scientific, San Jose, CA, USA) was used for the analysis.

### Biochemical Diagnoses

Biochemical diagnoses of IEMs were based on the results of MS/MS and/or GC-MS indicating obvious specific abnormal metabolites, in accordance with clinical data by physicians who specializing in IEMs.

### Genetic Analysis

Genomic DNA was extracted from peripheral blood leukocytes obtained from the patients using a QIAamp DNA Blood Mini Kit (Qiagen, Hilden, Germany). The lists of known inheritable genetic disease–related genes in the panels for captured and targeted next-generation sequencing were described previously (Yang et al., 2013; Chen et al., 2017). The amplified DNA was specifically enriched using a biotinylated capture probe (MyGenostics, MD, USA). Sanger sequencing was used to confirm the mutations. The polymerase chain reaction product was purified using solid phase reversible immobilization beads (Beckman Coulter, Inc.) according to the manufacturer’s protocol (He et al., 2010; Wu et al., 2011). The enrichment libraries were sequenced using an Illumina HiSeq 2000 sequencer. Short read mapping and alignment were performed using the Burrows Wheeler Aligner software.

### Clinical Data Analysis

The medical records of full-term MMA patients with definite genetic defect were analyzed, and the rates of each symptom were calculated to assess the difference between *MMUT* and *MMACHC* defects in NICU.

### Statistical Analysis

All analyses were done using SPSS software, version 19.0. The differences between rates of each symptoms were tested using *χ* ^2^ or Fisher exact tests, if appropriate. A *P* value less than 0.05 indicated a statistically significant difference.

### Availability of Data and Materials

The clinical data of the patient is available in the record room of the Seventh Medical Center of PLA General Hospital. The sequencing and mass spectrometry data is available in the laboratory of BaYi Children’s Hospital. Basic information of subjects and the genetic analysis and clinical symptom data of the patients are presented in the additional supporting files.

## Results

### Incidence and Composition of Inborn Errors of Metabolism in a Chinese Neonatal Intensive Care Unit

Among all 42,257 neonates admitted to the NICU, 66 patients were biochemically diagnosed with IEMs. This included 60 IEM patients of 26,750 full-term neonates and 6 IEM patients with IEMs of 15,507 premature neonates. The total prevalence in the NICU was 1:640 (66/42, 257), and the prevalence of full-term and premature cases was 1:446 (60/26, 750) and 1:2,584 (6/15, 507), respectively. Further, 60 full-term cases of IEM [(34 male and 26 female; median age 5 days (0–30 days), median gestation 39.21 weeks (37.00–42.00 weeks), and median birth weight 3,000 g (1,600–4,400 g)] included 46 cases of MMA (26 cases of isolated MMA and 20 cases of combined MMA/homocystinuria), 4 cases of propionic acidemia (PA), 3 cases of urea cycle disorders (UCD), 3 cases of maple syrup urine disease (MSUD), 2 cases of tyrosinemia (Tyr), 1 case of isovaleric acidemia (IVA), and 1 case of very long-chain acyl-CoA dehydrogenase deficiency (VLCADD). Also, six premature cases of IEM [five male and one female; median gestation 33.07 weeks (26.86–36.71 weeks), and median birth weight 2015 g (1,000–3,200 g)] included three cases of phenylketonuria (PKU) and one case each of MMA combined with Hcy, UCD, and glutaric aciduria type II (GAII) (see **Table 1**).

**Table 1:** Incidence of inborn errors of metabolism in neonatal intensive care unit population and case numbers of full-term and premature.

Disease		Incidence in NICU		Case numbers		Proportion (%)	
				**Full-term**	**Premature**		
MMA	Isolated	1:899	1:1,625	26	0	71.2	39.4
	With Hcy		1:2,012	20	1		31.8
PA		1:10,564		4	0	6.1	
UCD		1:10,564		3	1	6.1	
MSUD		1:14,086		3	0	4.5	
PKU		1:14,086		0	3	4.5	
Tyr		1:21,128		2	0	3.0	
IVA		1:42,257		1	0	1.5	
VLCADD		1:42,257		1	0	1.5	
GAII		1:42,257		0	1	1.5	
Total		1:640		60	6	100	

### A Genotypic Spectrum of Cases Biochemically Diagnosed With Inborn Errors of Metabolism in a Chinese Neonatal Intensive Care Unit

**Figure 1:**
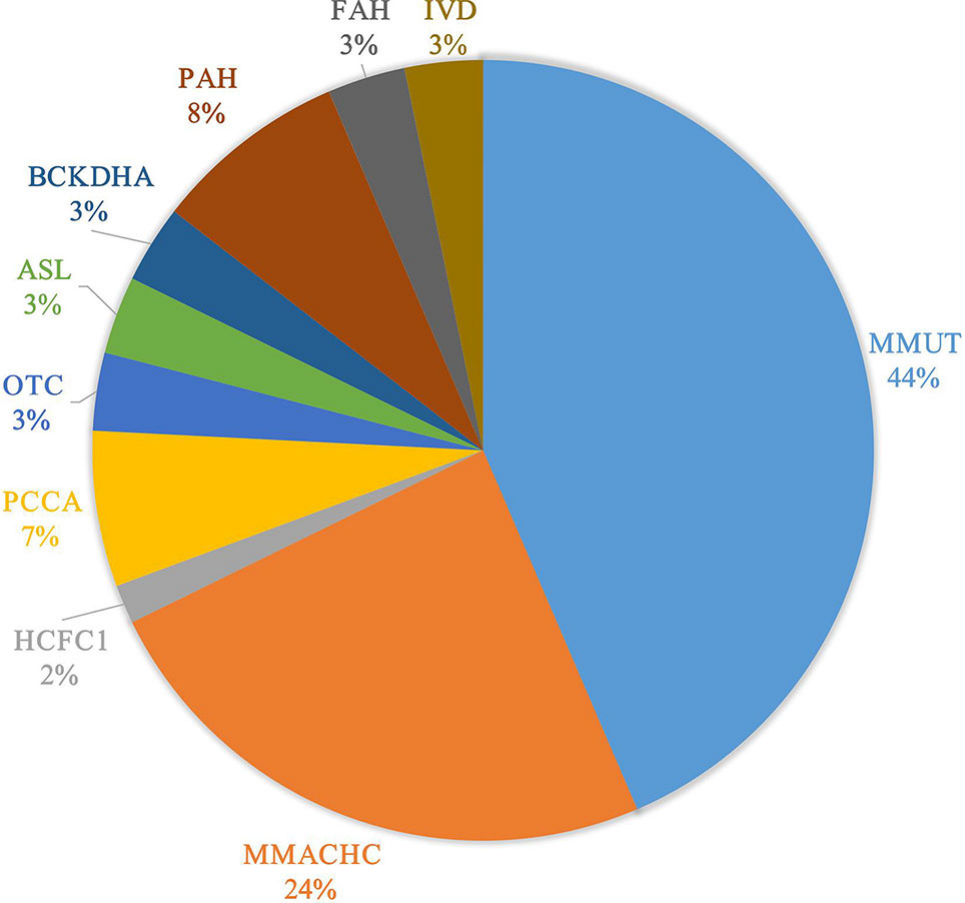
The genotypic spectrum in 49 cases of inborn errors of metabolism in Chinese neonatal intensive care unit.

Of 66 patients biochemically diagnosed with IEMs, 49 were sent for gene analysis, and 62 potentially pathogenic mutations in 10 genes were detected (**Table 2**). Twenty-one (33.9%) of these mutations were found unreported in this study (**Table 2**). Further, the eight types of IEMs associated with these mutations were completely consistent with the biochemical diagnosis. The distribution of mutations in different genes in 49 cases are shown in **Figure 1**. The most prevalently mutated gene was *MMUT* related to the occurrence of isolated MMA.

**Table 2:** Gene mutation spectrum of 49 cases of inborn errors of metabolism in Chinese neonatal intensive care unit.

Gene (cases)	Exons	Variant	Type of mutation	Reference PMID/ ClinVar ID※	Allele frequency % (n)	IEMs presentation
***MMUT* (20)**	2	c.323G > A (p.R108H)	Missense	11528502	10.0 (4)	Isolated methylmalonic acidurias
	3	c.729_730insTT (p.D244Lfs*39)	Frame shift	16281286	7.5 (3)	
	5	c.914T > C (p.L305S)	Missense	16281286	5.0 (2)	
	5	c.944dupT (p.Y316Lfs*11)	Frame shift	25863090	5.0 (2)	
	6	c.1106G > A (p.R369H)	Missense	9285782	5.0 (2)	
	6	c.1280G > A (p.G427D)	Missense	16281286	5.0 (2)	
	11	c.1874A > C (p.D625A)	Missense	30712249	5.0 (2)	
	2	c.91C > T (p.R31*)	Nonsense	16435223	2.5 (1)	
	2	c.322C > T (p.R108C)	Missense	16281286	2.5 (1)	
	3	c.424A > G (p.T142A)	Missense	19806564	2.5 (1)	
	3	c.683G > A (p.R228Q)	Missense	9554742	2.5 (1)	
	4	c.755dupA (p.H252Qfs*6)	Frame shift	23430940	2.5 (1)	
	6	c.1107dupT (p.T370Yfs*22)	Frame shift	30098236	2.5 (1)	
	IVS9	c.1677-1G > A	Splicing	16281286	2.5 (1)	
	10	c.1679G > A (p.C560Y)	Missense	16435223	2.5 (1)	
	12	c.2080C > T (p.R694W)	Missense	7912889	2.5 (1)	
	13	c.2179C > T (p.R727*)	Nonsense	16281286	2.5 (1)	
	3	c.428A > G (p.H143R)	Missense	Unreported (17113806)	2.5 (1)	
	3	c.470T > A (p.V157D)	Missense	Unreported (30712249)	2.5 (1)	
	4	c.861C > G (p.Y287*)	Nonsense	Unreported	2.5 (1)	
	4	c.877C > T (p.Q293*)	Nonsense	Unreported	2.5 (1)	
	6	c.1153_1154delTT (p.L385Afs*6)	Inframe deletion	Unreported	2.5 (1)	
	IVS9	c.1677-2A > G	Splicing	Unreported	2.5 (1)	
	10	c.1759T > C (p.Y587H)	Missense	Unreported (15643616, 22614770)	2.5 (1)	
	10	c.1787delA (p.E596Gfs*2)	Frame shift	Unreported	2.5 (1)	
	IVS11	c.1956+1del G	Splicing	Unreported	2.5 (1)	
	13	c.2194G > C (p.A732P)	Missense	Unreported	2.5 (1)	
***MMACHC* (17)**	4	c.609G > A (p.W203*)	Nonsense	16311595	38.2 (13)	Combined methylmalonic aciduria and homocystinuria, cblC type
	2	c.217C > T (p.R73*)	Nonsense	16311595	8.8 (3)	
	4	c.658_660delAAG (p.K220del)	Inframe deletion	16311595	8.8 (3)	
	2	c.271 dupA (p.R91Kfs*14)	Frame shift	16311595	5.9 (2)	
	4	c.567dupT (p.I190Yfs*13)	Frame shift	19370762	5.9 (2)	
	1	c.80A > G (p.Q27R)	Missense	16311595	2.9 (1)	
	3	c.315C > G (p.Y105X)	Nonsense	20631720	2.9 (1)	
	3	c.331C > T (p.R111*)	Nonsense	16311595	2.9 (1)	
	3	c.398_399delAA (p.Q133Rfs*5)	Frame shift	16311595	2.9 (1)	
	4	c.445_446del TG (p.C149Hfs*32)	Frame shift	26287336	2.9 (1)	
	4	c.616C > T (p.R206W)	Missense	16311595	2.9 (1)	
	4	c.666C > A (p.Y222*)	Nonsense	16311595	2.9 (1)	
	4	c.615C > A (p.Y205*)	Nonsense	558292※	2.9 (1)	
	4	c.658A > C (p.K220Q)	Missense	Unreported	5.9 (2)	
	4	c.511delG (p.V171Cfs*39)	Frame shift	Unreported	2.9 (1)	
***HCFC1* (1)**	18	c.4475C > G (p.P1492R)	Missense	373423※	100.0 (1)	Combined methylmalonic aciduria and homocystinuria, cblX type
***PCCA* (2)**	2	c.130_131insAT (p.C44Yfs*3)	Frame shift	Unreported	25.0 (1)	Propionic acidemia
	2	c.131G > T (p.C44F)	Missense	Unreported	25.0 (1)	
	19	c.1746+3G > C	Splicing	Unreported	25.0 (1)	
	—	exon 7-9 deletion	Large deletion	Unreported	25.0 (1)	
***OTC* (2)**	2	c.214G > T (p.E72*)	Nonsense	Unreported	50.0 (1)	Urea cycle disorder (ornithine transcarbamylase deficiency)
	10	c.1016T > G (p.V339G)	Missense	25932215	50.0 (1)	
***ASL* (1)**	8	c.544C > T (p.R182*)	Nonsense	17326097	50.0 (1)	Urea cycle disorder (argininosuccinic aciduria)
	10	c.706C > T (p.R236W)	Missense	17326097	50.0 (1)	
***BCKDHA* (1)**	1	c.108+4A > G	Splicing	Unreported	50.0 (1)	Maple syrup urine disease
	2	c.117dupC (p.R40Qfs*11)	Frame shift	8037208	50.0 (1)	
***PAH* (3)**	6	c.611A > G (p.Y204C)	Missense	23430918	16.7 (1)	Phenylketonuria
	6	c.688G > A (p.V230I)	Missense	8268925	16.7 (1)	
	7	c.728G > A (p.R243Q)	Missense	2071149	33.3 (2)	
	7	c.764T > C (p.L255S)	Missense	2014802	16.7 (1)	
	11	c.1199G > A (p.R400K)	Missense	16256386	16.7 (1)	
***FAH* (1)**	6	c.494C > T (p.S165F)	Missense	Unreported	50.0 (1)	Tyrosinemia type 1
	9	c.782 C > T (p.P261L)	Missense	9633815	50.0 (1)	
***IVD* (1)**	1	c.134T > G (p.L45R)	Missense	Unreported (2063866)	50.0 (1)	Isovaleric acidemia
	12	Exon 12 deletion	Large deletion	Unreported	50.0 (1)	

Under the column “Reference,” the PMID in () references for a different amino acid change in previously reported positions; ※ means the variant was unreported in PubMed while has been annotated in ClinVar.

In 38 MMA cases who underwent genetic analysis, 43 potentially pathogenic mutations were found in three genes (including *MMUT*, *MMACHC*, and *HCFC1*) (**Table 2**). Among 27 *MMUT* mutations, the most common mutation was c.323G > A (p.R108H) in exon 2 which detected in four patients, and most of these mutations (74.1%, 20/27) were identified only in isolated cases (**Supplementary Table 2**).

Fifteen *MMACHC* mutations were identified in 17 cases, and the most common mutation was c.609 G > A (p.W203*), which occurred in 38.2% of all disease alleles in exon 4. The exon 4 of *MMACHC* in this study encompasses the largest number of mutations (66.7%, 10/15) which including two unreported ones (**Table 2**).

### Clinical Characteristics of Neonates With Methylmalonic Acidemia in the Neonatal Intensive Care Unit

There were 20 *MMUT* and 17 *MMACHC* defects in 38 MMA neonates (80.8%, 38/47) that performed genetic analysis. The differences in clinical characteristics of the above two in the NICU were analyzed, and the only case of premature birth with *MMACHC* defect was excluded because multiple clinical symptoms caused by premature birth are easily confused with MMA.

When admitted to the NICU, 11 cases among the 16 full-term patients with *MMACHC* mutations showed clinical symptoms suspected of IEMs. Further, 4 of 11 were early onset within 0–7 days after birth. According to the results of vitamin B12 loading test, all patients with *MMACHC* mutations were diagnosed with vitamin B12 responsiveness. However, four deaths still occurred during an acute metabolic crisis in the NICU (**Supplementary Table 3**). The common symptoms of 16 *MMACHC* cases in the NICU included respiratory distress/pneumonia (15, 93.8%), poor response or milk refusal (11, 68.8%), metabolic acidosis (9, 56.2%), anemia (9, 56.2%) and encephalopathy (9, 56.2%) (**Table 3**).

**Table 3:** Manifestations of full-term methylmalonic acidemia cases with *MMU* *T* (20) and *MMACHC*(16) defects in neonatal intensive care unit.

Manifestations	*MMUT*% (n=20)	*MMACHC*% (n=16)	*p* value
Responsiveness to VitB12	**20.0 (4)**	**100 (16)**	**0.0000**
Early onset in 0-7 days	**90.0 (18)**	**25.0 (4)**	**0.0001**
Metabolic acidosis	**100.0 (20)**	**56.2 (9)**	**0.0014**
Electrolyte disturbances	**85.0 (17)**	**31.2 (5)**	**0.0017**
Neonatal death	**70.0 (14)**	**25.0 (4)**	**0.0176**
Coagulant function abnormality	**60.0 (12)**	**25.0 (4)**	**0.0485**
Glucose metabolism dysfunction	**45.0 (9)**	**12.5 (2)**	**0.0671**
Poor response or milk refusal	**95.0 (19)**	**68.8 (11)**	**0.0689**
Hyperammonemia	**20.0 (4)**	**0 (0)**	**0.1131**
Jaundice/liver failure	**55.0 (11)**	**31.2 (5)**	**0.1914**
Anemia	**35.0 (7)**	**56.2 (9)**	**0.3128**
Progressive encephalopathy	**35.0 (7)**	**56.2 (9)**	**0.3128**
Respiratory distress/pneumonia	**80.0 (16)**	**93.8 (15)**	**0.3549**
Skin lesions	**10.0 (2)**	**25.0 (4)**	**0.3738**
Respiratory failure	**15.0 (3)**	**6.2 (1)**	**0.6129**
Congenital heart disease	**15.0 (3)**	**12.5 (2)**	**1.0000**
Infection/sepsis	**15.0 (3)**	**18.8 (3)**	**1.0000**
Mature low birth weight	**15.0 (3)**	**18.8 (3)**	**1.0000**
Myocardial damage	**30.0 (6)**	**25.0 (4)**	**1.0000**
Renal injury	**10.0 (2)**	**6.2 (1)**	**1.0000**
Seizures	**20.0 (4)**	**18.8 (3)**	**1.0000**

All 20 cases with *MMUT* mutations presented metabolic symptoms suspected of IEMs upon admission to the NICU, and 90.0% (18/20) developed these symptoms within 0–7 days after birth. Sixteen cases of 20 showed non-responsiveness to vitamin B12. Among non-responsiveness cases, 14 died during an acute metabolic crisis in the NICU; while four patients who diagnosed with vitamin B12 responsiveness were discharged with a better health condition. The common symptoms of 20 *MMUT* cases in the neonatal period included metabolic acidosis (20, 100%), poor response or milk refusal (19, 95.0%), electrolyte disturbances (17, 85.0%), respiratory distress/pneumonia (16, 80.0%), coagulant function abnormality (12, 60.0%), and jaundice/liver failure (11, 55.0%) (**Table 3**).

## Discussion

In this study, we reported the incidence of IEMs in the NICU population including full-term and preterm neonates for 7 years in north China mainland. The disease spectrum of IEMs in a Chinese NICU was obtained based on the data of 42,257 newborns. The prevalence of IEMs in our NICU as 1:640, while the data reported were higher (1–2%) based on much fewer cases (Couce et al., 2011; Tu et al., 2012). This difference might have originated from the distinct standards of enrollment to the NICU. The reported high incidence of IEMs comes from survey of elder infants or children with a higher risk of metabolic disorders, while the present study focused on a population of neonates within 1 month of admission to the NICU. High proportion of premature neonates (36.7%) and more accurate top limit time of admission are the hallmarks of our survey. The incidence of full-term neonates was 1:446 in this study, which was approximately six times more than 1:2,584 in premature neonates and 7–10 times more than 1:3,065 to 1:4,300 in normal newborns (Frazier et al., 2006; Shi et al., 2012; Lim et al., 2014; Therrell et al., 2015; Wilcken and Wiley, 2015). In addition, full-term neonates accounted for 90.9% (60/66) of all IEM cases in a Chinese NICU population with the ratio of full-term and premature infants as 1.72:1. Despite several IEMs are known to be associated with premature birth (Carrillo-Carrasco et al., 2012) (Vianey-Saban et al., 2016) (Guibaud et al., 2017), no direct association between IEMs and premature birth can be concluded when reviewing the clinical history of all six cases of premature in our study.

In the spectrum of diseases that we obtained, a total of 53 cases in four kinds of organic aciduria (MMA, PA, IVA, and GAII) accounted for 80.3%, 12 cases in 5 kinds of amino acidic disorders accounted for 18.2%, and 1 case of fatty acid oxidation disorders accounted for the remaining 1.5%. Further, 93.9% (62/66) of these cases belong to the group that caused endogenous intoxication (including MMA, PA, UCD, MSUD, TyrI, IVA, GAII) which can display early onset in neonatal period (Saudubray et al., 2002) and this ratio of endogenous intoxication group was more in full-term infants with 98.3% (59/60), while the dominating type of IEM among preterm patients was PKU (50%, 3/6), a typical kind of late-onset IEMs. However, the most common IEM among full-term neonates was MMA (including isolated type and combined type with Hcy), which was also the most common IEM in all NICU cases, accounting for 71.2% (isolated type as 39.4%, combined type with Hcy as 31.8%) in disease spectrum. PKU and MMA ranked the top 2 of all kinds of IEMs diagnosed in the Chinese population, the proportion of MMA are between 10.2 and 36.6%. (Han et al., 2008; Shi et al., 2012; Guo et al., 2018). Intriguingly, the proportion of MMA was dramatically higher in our NICU. Meanwhile, the incidence of MMA in NBS was reported to be 1:3,920 in Shandong, 1:6,032 in Henan, 1:26,000 in Shanghai/Beijing, and 1:46,532 in Zhejiang, respectively (Hong et al., 2017; Zhou et al., 2018). Strikingly, this ratio in our NICU was 1:899.

The higher incidence of IEMs in sick full-term as opposed to premature newborns doesn’t indicate that the full term neonates are more prone to develop IEMs, rather than IEMs were not a frequent cause of premature births, at least in this cohort. Our result likely reflects the fact that maternal and placental metabolism protects infants with IEMs until birth and they present soon after delivery and exposure to stress and feeds. The higher incidence of IEMs in the NICU cohort compared to normal/NBS cohorts reflects the disease burden of early onset intoxication disorders that typically present with severe metabolic ketoacidosis and hyperammonemia in the first hours/days of life. And the detection of more PKU cases in the premature cohort doesn’t mean that PKU caused the prematurity, as it is a more common IEM.

Late onset IEMs like PKU, VLCADD often do not have clinical manifestations in the neonatal period. We reviewed these cases of late onset IEMs found in our study, they admitted to NICU because of pneumonia, infection, and other common causes, without obvious symptoms suspected IEMs. These cases have been diagnosis of IEMs by MS/MS screening much earlier before symptoms appear and were immediately subjected to treatment, indicating the advantages of MS/MS screening in early intervention of IEMs.

Detailed analysis of hotspot mutant genes was also conducted in this integrative study. The *MMACHC* mutations were reported to account for the majority of Chinese MMA cases (Guo et al., 2018; Liu et al., 2018); while in the spectrum of IEM genes in NICU we obtained, *MMUT* mutations were more common. This might because of worsened clinical manifestations of *MMUT* defect in the neonatal period. Moreover, many neonatal deaths displaying this defect had not experienced diagnosis, leading to loss of the statistic reports from surviving cases of elder children. In previous reports, the two most common mutations of *MMUT* were c.729_730insTT and c.323G > A among Chinese patients with different ages of onset ranging from newborn to 8 years (Han et al., 2015). However, in our study based on neonatal patients, the proportion of c.323G > A was slightly higher than c.729_730insTT (**Table 2**). A total of 20 mutations accounting for half of the disease alleles were identified only in one allele, and a large proportion of new mutations (37.0%) and compound heterozygotes (100%) were found, demonstrating the highly pleomorphic nature and genetic heterogeneity of *MMUT* gene as reported. Parallelly, among 15 different *MMACHC* mutations identified in 17 cases, the most common mutation was c.609 G > A (p.W203*) in exon 4, which occurred in 38.2% of all disease alleles. Strikingly, we found that the mutations of *MMACHC* in a Chinese NICU were concentrated at the C-terminal of exon 4 (9 in 15). Furthermore, a hotspot mutation in c.658 (p.220) was found in highly conserved regions of homologous sequences on exon 4 with a frequency of 14.7%.

The limited number of patients included in this study, the high frequency of compound heterozygotes, and the lack of enzymatic studies all rendering the assessment the precise relationship between gene mutations and clinical manifestations difficult. An *MMACHC* mutation [c.609G > A (p.W203*)], which was reported to be the most common hot site associated with early onset (Yu et al., 2015), was found in the homozygous state in two cases (cases 21 and 22 in **Supplementary Table 3**) of the study. Both cases had severe neonatal symptoms such as roaring production of lactic acid. One died of metabolic crisis, and the other was discharged from the NICU in a good condition after active treatment in NICU. In addition, an unreported missense mutation of *MMACHC* as c.658A > C (p.K220Q) was homozygous in case 23 (**Supplementary Table 2**). Case 23 was admitted due to poor appetite, dyspnea, and fever at 28 days of age. The clinical symptoms of this case were mild without typical lactic acid, intoxication, and metabolic disorders of electrolytes and blood sugar; also, vitamin B12 treatment was found to be effective in this case. Two mutations in c.658 (located at p.220), including c.658A > C, c.658_660delAAG, accounted for 14.7% in all disease alleles of *MMACHC*, and c.658_660delAAG was reported to be common in the Chinese population and associated with early onset (Zhou et al., 2018). We speculated that the c.658 is a mutation hotspot in Chinese neonates.

Synergistically, the correlation between clinical features and *MMUT* and *MMACHC* defects in full-term neonates were calculated in this study. The number of patients with early onset in PN0–PN7 days, metabolic acidosis, electrolyte disturbances, coagulant function abnormality, and neonatal death were significantly larger in *MMUT* defect than in *MMACHC* defect, while the quantity of cases responded to vitamin B12 treatment with significantly improved biochemical indicators was much smaller in *MMUT* defect than in *MMACHC* defect (**Table 3**). The mortality of *MMUT* cases (70.0%) was much higher than the total mortality of IEMs in the NICU (53.0%, 35 deaths in 66 cases were all attributed to the endogenous toxic type of IEMs). On the other side, the mortality of *MMACHC* defects was only 25.0%. The dampened survival rate in these cases of IEMs was mostly due to the reluctance of the parents for accepting the fit-time treatments after informing the result of biochemical diagnosis. Moreover, 21.2% (14/66) of diagnosed IEM cases had a history of IEMs or neonatal death in siblings, but their parents had no prenatal counseling or genetic testing. Therefore, it is necessary to improve parents’ cognition of IEMs and the rate of genetic diagnosis of probands.

To sum up, we screened 42,257 NICU newborns by mass spectrometry and diagnosed 66 cases of IEMs. We found in Chinese NICU the incidence of IEMs was 1:640, and the incidence in full-term neonates (1:446) was significantly higher than that in premature ones (1:2,584). The most common IEM in total and full-term infants was MMA (in particular the isolated type), while in premature infants was PKU. The higher incidence and the overwhelming proportion (> 90%) of endogenous intoxication type of IEMs in disease spectrum we obtained reflects the disease burden of these disorders in NICU. We found 62 potentially pathogenic mutations in 10 genes of IEMs in genetic analysis, 33.9% (21) of them were novel. The obtained spectra of mutation genes indicated that the most prevalent defect gene was *MMUT*. Our results enriched the clinical characteristics and genetic data of neonatal IEMs and provided diagnostic clues and therapeutic insights for neonatal pediatricians.

## Data Availability Statement

The clinical data of the patient is available in the record room of the Seventh Medical Center of PLA General Hospital. The sequencing and mass spectrometry data is available in the laboratory of BaYi Children’s Hospital. Basic information of subjects and the genetic analysis and clinical symptom data of the patients are presented in the additional supporting files. The data are not publicly available due to them containing information that could compromise research participant privacy/consent.

## Ethics Statement

This study was carried out in accordance with the recommendations of “Medical ethics committee of PLA general hospital” with written informed consent from all the patients’ guardians for experimentation. All procedures followed were in accordance with the ethical standards of “Medical ethics committee of PLA general hospital” and with the Helsinki Declaration.

## Author Contributions

WZ and YY performed and interpreted the results of DBS and urine screening of IEMs, analyzed the patient’s data, and wrote the manuscript. WP and YC analyzed the genetic data and the structural of the protein. JC and YM performed the medical assessment, provided the clinical data, assisted with the data analysis, and created the figures. LY, XW, and YL involved in sample collection, implementation of DBS and urine screening, and drafting the manuscript. YW and ZF provided guidance in patient management, participated in the manuscript preparation and revision. All authors have read and approved the final manuscript.

## Funding

This work was supported by the National Key R&D Program of China (2017YFC1001700) and the National Natural Science Foundation of China (31100603).

## Conflict of Interest

The authors declare that the research was conducted in the absence of any commercial or financial relationships that could be construed as a potential conflict of interest.
