# Supplementary material for: A 7-Year Report of Spectrum of Inborn Errors of Metabolism on Full-Term and Premature Infants in a Chinese Neonatal Intensive Care Unit
Source: Front Genet. 2020 Jan 10;10:1302. doi: 10.3389/fgene.2019.01302 (PMC6967400; doi:10.3389/fgene.2019.01302)
Supplement: Supplementary file 1 [file DataSheet_1.docx]

**Supplementary Table 1. Basic information of full term and premature neonates in our NICU population.**

| NICU neonates  （42, 257）  Basic Information | Full term  (≥ 37wk)  （n=26, 750) | Premature  (< 37wk)  (n=15, 507) | *p* value |
| --- | --- | --- | --- |
| Gestational Age (w) | 39.24tiona | 32.40tiona | < 0.05 |
| Days of admission（d） | 9.45 of ad | 0 |  |
| Birth Weight（g） | 3060.67eightss | 2016.67eightss |  |
| Gender（M/F） | 1.41/ 1 | 1.43/ 1 | > 0.05 |

**Supplementary Table 2. Genetic analysis of 49 Biochemical diagnosis cases.** M, male; F, female; * Premature case; ※ClinVar ID.

| Case No. | Gender | Biochemical diagnosis | Gene | RefSeq | Genotype | cDNA  change | Protein  change | Reference  PMID/ ClinVar ID※ |
| --- | --- | --- | --- | --- | --- | --- | --- | --- |
| 1 | F | MMA (isolated) | *MMUT* | NM_000255.3 | Comp Het | c.323G>A c.914T>C | p.R108H  p.L305S | 11528502  16281286 |
| 2 | M | MMA (isolated) | *MMUT* | NM_000255.3 | Comp Het | c.323G>A c.1759T>C | p.R108H  p.Y587H | 11528502  Unreported (15643616, 22614770) |
| 3 | F | MMA (isolated) | *MMUT* | NM_000255.3 | Comp Het | c.323G>A c.2179C>T | p.R108H  p.R727* | 11528502  16281286 |
| 4 | M | MMA (isolated) | *MMUT* | NM_000255.3 | Comp Het | c.729_730insTT  c.424A>G | p.D244Lfs*39  p.T142A | 16281286  19806564 |
| 5 | M | MMA (isolated) | *MMUT* | NM_000255.3 | Comp Het | c.729_730insTT  c.1280G>A | p.D244Lfs*39 p.G427D | 16281286  16281286 |
| 6 | F | MMA (isolated) | *MMUT* | NM_000255.3 | Comp Het | c.729_730insTT c.1956+1del G | p.D244Lfs*39  Mis-splicing | 16281286  Unreported |
| 7 | F | MMA (isolated) | *MMUT* | NM_000255.3 | Comp Het | c.914T>C  c.683G>A | p.L305S  p.R228Q | 16281286  9554742 |
| 8 | M | MMA (isolated) | *MMUT* | NM_000255.3 | Comp Het | c.944dupT  c.428A>G | p.Y316Lfs*11 p.H143R | 25863090  Unreported (17113806) |
| 9 | F | MMA (isolated) | *MMUT* | NM_000255.3 | Comp Het | c.944dupT  c.2194G>C | p.Y316Lfs*11  p.A732P | 25863090  Unreported |
| 10 | M | MMA (isolated) | *MMUT* | NM_000255.3 | Comp Het | c.1106G>A  c.755dupA | p.R369H  p.H252Qfs*6 | 9285782  23430940 |
| 11 | F | MMA (isolated) | *MMUT* | NM_000255.3 | Comp Het | c.1106G>A  c.1787delA | p.R369H  p.E596Gfs*2 | 9285782  Unreported |
| 12 | M | MMA (isolated) | *MMUT* | NM_000255.3 | Comp Het | c.1679G>A  c.1677-1G>A | p.C560Y  Mis-splicing | 16435223  16281286 |
| 13 | M | MMA (isolated) | *MMUT* | NM_000255.3 | Comp Het | c.1677-2A>G  c.91C>T | Mis-splicing  p.R31* | Unreported  16435223 |

| 14 | M | MMA (isolated) | *MMUT* | NM_000255.3 | Comp Het | c.877C>T  c.861C>G | p.Q293X  p.Y287X | Unreported  Unreported |
| --- | --- | --- | --- | --- | --- | --- | --- | --- |
| 15 | F | MMA (isolated) | *MMUT* | NM_000255.3 | Comp Het | c.1874A>C  c.470T>A | p.D625A  p.V157D | 30712249  Unreported (30712249) |
| 16 | F | MMA (isolated) | *MMUT* | NM_000255.3 | Comp Het | c.1280G>A; c.322C>T | p.G427D  p.R108C | 16281286  16281286 |
| 17 | M | MMA (isolated) | *MMUT* | NM_000255.3 | Comp Het | c.1107dupT  c.1874A>C | p.T370Yfs*22 p.D625A | 30098236  30712249 |
| 18 | F | MMA (isolated) | *MMUT* | NM_000255.3 | Carrier | c.323G>A | p.R108H | 11528502 |
| 19 | M | MMA (isolated) | *MMUT* | NM_000255.3 | Carrier | c.2080C>T | p.R694W | 7912889 |
| 20 | M | MMA (isolated) | *MMUT* | NM_000255.3 | Carrier | c.1153_1154delTT | p.L385Afs*6 | Unreported |
| 21 | F | MMA  (with Hcy) | *MMACHC* | NM_015506.2 | Hom | c.609G>A | p.W203* | 16311595 |
| 22 | M | MMA  (with Hcy) | *MMACHC* | NM_015506.2 | Hom | c.609G>A | p.W203* | 16311595 |
| 23 | M | MMA  (with Hcy) | *MMACHC* | NM_015506.2 | Hom | c.658A>C | p.K220Q | Unreported |
| 24 | M | MMA  (with Hcy) | *MMACHC* | NM_015506.2 | Comp Het | c.609G>A  c.217C>T | p.W203*  p. R73* | 16311595  16311595 |
| 25 | F | MMA  (with Hcy) | *MMACHC* | NM_015506.2 | Comp Het | c.609G>A  c.217C>T | p.W203*  p. R73* | 16311595  16311595 |
| 26 | F | MMA  (with Hcy) | *MMACHC* | NM_015506.2 | Comp Het | c.609G>A  c.658_660delAAG | p.W203*  p.K220del | 16311595  16311595 |
| 27 | M | MMA  (with Hcy) | *MMACHC* | NM_015506.2 | Comp Het | c.609G>A  c.658_660delAAG | p.W203*  p.K220del | 16311595  16311595 |
| 28 | M | MMA  (with Hcy) | *MMACHC* | NM_015506.2 | Comp Het | c.271 dupA  c.609G>A | p.R91Kfs*14  p.W203* | 16311595  16311595 |
| 29 | F | MMA  (with Hcy) | *MMACHC* | NM_015506.2 | Comp Het | c.609G>A  c.567dupT | p.W203*  p.I190Yfs*13 | 16311595  19370762 |
| 30 | F | MMA  (with Hcy) | *MMACHC* | NM_015506.2 | Comp Het | c.315C>G  c.609G>A | p.Y105*  p.W203* | 20631720  16311595 |
| 31* | M | MMA  (with Hcy) | *MMACHC* | NM_015506.2 | Comp Het | c.609G>A  c.666C>A | p.W203*  p.Y222* | 16311595  16311595 |
| 32 | M | MMA  (with Hcy) | *MMACHC* | NM_015506.2 | Comp Het | c.609G>A  c.80A>G | p.W203*  p. Q27R | 16311595  16311595 |
| 33 | M | MMA  (with Hcy) | *MMACHC* | NM_015506.2 | Comp Het | c.217C>T  c.445_446del TG | p.R73*  p.C149Hfs*32 | 16311595  26287336 |
| 34 | M | MMA  (with Hcy) | *MMACHC* | NM_015506.2 | Comp Het | c.271 dupA  c.398_399delAA | p.R91Kfs*14  p.Q133Rfs*5 | 16311595  16311595 |
| 35 | M | MMA  (with Hcy) | *MMACHC* | NM_015506.2 | Comp Het | c.567dupT  c.658_660delAAG | p.I190Yfs*13  p.K220del | 19370762  16311595 |
| 36 | M | MMA  (with Hcy) | *MMACHC* | NM_015506.2 | Comp Het | c.616C>T  c.615C>A | p.R206W  p.Y205* | 16311595  558292※ |
| 37 | F | MMA  (with Hcy) | *MMACHC* | NM_015506.2 | Comp Het | c.331C>T  c.511delG | p.R111* p.V171Cfs*39 | 16311595  Unreported |
| 38 | M | MMA  (with Hcy) | *HCFC1* | NM_005334 | Hemi | c.4475C>G | p.P1492R | 373423※ |
| 39 | M | PA | *PCCA* | NM_000282 | Comp Het | c.130_131insAT  c.131G>T | p.C44Yfs*3  p.C44F | Unreported  Unreported |
| 40 | F | PA | *PCCA* | NM_000282 | Comp Het | c.1746+3G>C  exon 7-9 deletion | Mis-splicing  - | Unreported  Unreported |
| 41 | M | UCD | *OTC* | NM_000531 | Hemi | c.214G>T | p.E72* | Unreported |
| 42 | M | UCD | *OTC* | NM_000531 | Hemi | c.1016T>G | p.V339G | 25932215 |
| 43 | F | UCD | *ASL* | NM_000048.3 | Comp Het | c.706C>T c.544C>T | p.R236W  p.R182* | 17326097  17326097 |
| 44 | F | MSUD | *BCKDHA* | NM_000709 | Comp Het | c.108+4A>G  c.117dupC | Mis-splicing  p.R40Qfs*11 | Unreported  8037208 |
| 45* | M | PKU | *PAH* | NM_000277.1 | Comp Het | c.611A>G  c.764T>C | p.Y204C  p.L255S | 23430918  2014802 |
| 46* | F | PKU | *PAH* | NM_000277.1 | Comp Het | c.728G>A  c.688G>A | p.R243Q  p.V230I | 2071149  8268925 |
| 47* | F | PKU | *PAH* | NM_000277.1 | Comp Het | c.1199G>A c.728G>A | p.R400K  p.R243Q | 16256386  2071149 |
| 48 | F | TYR | *FAH* | NM_000137 | Comp Het | c.782 C>T  c.494C>T | p.P261L  p.S165F | 9633815  Unreported |
| 49 | F | IVA | *IVD* | NM_002225.3 | Comp Het | c.134T>G;  exon 12 deletion | p.L45R  - | Unreported (2063866)  Unreported |

**Note: Under the column “Reference”, the PMID in ( ) references for a different amino acid change in previously reported positions;**※ **means the variant was unreported in PubMed while has been annotated in ClinVar.**

**Supplementary Table 3. The clinical** **characteristics of MMA cases with *MUT* (20), *MMACHC*(17) and *HCFC1*(1) defects in our NICU.**

△ Unreported; * Premature case.

| **Case No.** | **Gene** | **Genotype** | **Mature low birth weight** | **Early onset in 0-7 days** | **Poor response or milk refusal** | **Respiratory distress/****pneumonia** | **[Metabolic acidosis](javascript:;)** | **Electrolyte disturbances** | **Hyperammonemia** | **Glucose metabolism dysfunction** | **Coagulant function abnormality** | **Congenital heart disease** | **Myocardial damage** | **Seizures** | **Jaundice** | **Hypotonia** | **Encephalopathy** | **Skin lesions** | **Anemia** | **Responsiveness to VitB12** | **Neonatal Death** | |
| --- | --- | --- | --- | --- | --- | --- | --- | --- | --- | --- | --- | --- | --- | --- | --- | --- | --- | --- | --- | --- | --- | --- |
| 1 | ***MUT*** | c.323G>A; c.914T>C | N | Y | Y | Y | Y | Y | N | N | Y | Y | Y | N | Y | N | N | N | N | N | Y |  |
| 2 |  | c.323G>A; c.1759T>C^△^ | N | Y | Y | N | Y | N | N | N | Y | N | N | Y | N | N | Y | N | Y | N | Y |  |
| 3 |  | c.323G>A; c.2179C>T | N | Y | Y | Y | Y | Y | N | N | Y | N | Y | N | Y | N | N | N | N | Y | N |  |
| 4 |  | c.729_730insTT; c.424A>G | N | Y | Y | Y | Y | Y | N | N | Y | N | Y | N | N | N | Y | N | Y | Y | N |  |
| 5 |  | c.729_730insTT; c.1280G>A | N | Y | Y | Y | Y | N | N | Y | N | N | N | N | Y | N | N | N | Y | N | N |  |
| 6 |  | c.729_730insTT; c.1956+1del G^△^ | N | Y | Y | N | Y | Y | Y | Y | N | N | Y | Y | N | N | N | N | N | N | Y |  |
| 7 |  | c.914T>C; c.683G>A | Y | Y | Y | N | Y | Y | N | N | Y | N | N | N | Y | N | Y | N | N | Y | N |  |
| 8 |  | c.944dupT; c.428A>G^△^ | N | Y | Y | Y | Y | Y | Y | Y | Y | N | Y | N | Y | N | N | N | Y | N | Y |  |
| 9 |  | c.944dupT; c.2194G>C^△^ | N | Y | Y | Y | Y | Y | N | N | N | N | N | N | Y | N | N | N | N | N | Y |  |
| 10 |  | c.1106G>A; c.755dupA | N | Y | Y | Y | Y | Y | N | N | N | N | N | N | Y | N | N | N | N | N | Y |  |
| 11 |  | c.1106G>A; c.1787delA^△^ | N | Y | Y | Y | Y | Y | N | N | Y | N | N | N | Y | N | Y | N | N | N | Y |  |
| 12 |  | c.1679G>A; c.1677-1G>A | N | Y | Y | Y | Y | Y | N | Y | Y | N | Y | N | N | N | N | N | Y | N | Y |  |
| 13 |  | c.1677-2A>G^△^; c.91C>T | N | Y | Y | Y | Y | Y | N | N | Y | N | N | N | N | N | Y | N | N | N | Y |  |
| 14 |  | c.877C>T^△^; c.861C>G^△^ | N | Y | Y | Y | Y | Y | N | Y | Y | N | N | N | Y | N | N | N | Y | N | Y |  |
| 15 |  | c.1874A>C; c.470T>A^△^ | N | Y | Y | Y | Y | Y | N | Y | N | Y | N | N | N | N | N | Y | N | N | Y |  |
| 16 |  | c.1280G>A; c.322C>T | N | N | N | N | Y | N | N | N | N | N | N | Y | N | N | N | N | N | Y | N |  |
| 17 |  | c.1107dupT; c.1874A>C | N | Y | Y | Y | Y | Y | Y | Y | Y | N | N | Y | N | N | Y | Y | N | N | Y |  |
| 18 |  | c.G323A | Y | Y | Y | Y | Y | Y | Y | Y | N | Y | N | N | N | N | N | N | N | N | Y |  |
| 19 |  | c.2080C>T | N | N | Y | Y | Y | Y | N | N | Y | N | N | N | Y | Y | Y | N | Y | N | N |  |
| 20 |  | c.1153_1154delTT^△^ | Y | Y | Y | Y | Y | Y | N | Y | N | N | N | N | Y | N | N | N | N | N | Y |  |
| 21 | ***MMACHC*** | c.609G>A (Hom) | N | Y | Y | Y | Y | N | N | N | N | N | N | N | Y | Y | N | N | N | Y | Y | |
| 22 |  | c.609G>A (Hom) | N | Y | N | Y | Y | N | N | N | N | N | Y | N | Y | N | Y | N | Y | Y | N | |
| 23 |  | c.658A>C^△^ (Hom) | N | N | Y | Y | N | N | N | N | N | N | N | N | N | N | Y | N | Y | Y | N | |
| 24 |  | c.609G>A; c.217C>T | N | N | Y | Y | Y | Y | N | Y | N | Y | N | N | N | N | N | Y | Y | Y | Y | |
| 25 |  | c.609G>A; c.217C>T | Y | N | N | Y | Y | Y | N | N | Y | Y | N | N | N | N | Y | Y | Y | Y | Y | |
| 26 |  | c.609G>A; c.658_660delAAG | N | N | N | N | N | N | N | N | N | N | N | Y | N | Y | N | N | N | Y | N | |
| 27 |  | c.609G>A; c.658_660delAAG | N | N | Y | Y | Y | N | N | N | Y | N | Y | N | N | N | N | N | Y | Y | Y | |
| 28 |  | c.271dupA; c.609G>A | N | N | Y | Y | N | N | N | N | N | N | Y | N | Y | N | N | N | Y | Y | N | |
| 29 |  | c.609G>A; c.567dupT | N | N | Y | Y | N | N | N | N | N | N | N | Y | N | N | Y | N | N | Y | N | |
| 30 |  | c.315C>G; c.609G>A | Y | N | Y | Y | Y | Y | N | N | Y | N | N | N | N | N | N | Y | Y | Y | N | |
| 31 |  | c.609G>A; c.666C>A | N | N | N | Y | N | N | N | N | N | N | N | N | Y | N | Y | N | N | Y | N | |
| 32* |  | c.609G>A; c.80A>G |  |  |  |  |  |  |  |  |  |  |  |  |  |  |  |  |  |  |  |  |
| 33 |  | c.217C>T;  c.445_446del TG | N | N | Y | Y | N | N | N | N | N | N | N | N | N | N | Y | Y | Y | Y | N |  |
| 34 |  | c.271dupA; c.398_399delAA | Y | N | Y | Y | Y | Y | N | N | Y | N | N | N | N | N | Y | N | Y | Y | N |  |
| 35 |  | c.567dupT; c.656_658delAGA | N | Y | N | Y | N | N | N | N | N | N | Y | Y | Y | N | N | N | N | Y | N |  |
| 36 |  | c.616C>T; c.615C>A | N | Y | Y | Y | Y | N | N | N | N | N | N | N | N | N | Y | N | N | Y | N |  |
| 37 |  | c.331C>T; c.511delG^△^ | N | N | Y | Y | Y | Y | N | Y | N | N | N | N | N | N | Y | N | N | Y | N |  |
| 38 | ***HCFC1*** | c.4475C>G | N | Y | Y | Y | Y | Y | N | N | Y | Y | N | N | N | Y | N | N | Y | Y | Y |  |
